# Sex dimorphism in the effect and predictors of weight loss after sleeve gastrectomy

**DOI:** 10.3389/fendo.2023.1333051

**Published:** 2024-01-10

**Authors:** Jiaxin Shu, Tao Zhu, Sisi Xiong, Teng Liu, Yian Zhao, Xin Huang, Shaozhuang Liu

**Affiliations:** ^1^ Division of Bariatric and Metabolic Surgery, Department of General Surgery, Qilu Hospital of Shandong University, Jinan, China; ^2^ Department of Surgery, First Clinical College, Shandong University, Jinan, China

**Keywords:** sleeve gastrectomy, weight loss, sex dimorphism, predictor, nomogram

## Abstract

**Background:**

No sex-specific guidelines for surgical anti-obesity strategies have been proposed, partially due to the controversy regarding sex-related differences in weight loss after bariatric metabolic surgery.

**Objectives:**

To explore sex dimorphism in the effect and predictors of weight loss after sleeve gastrectomy (SG), thereby providing clinical evidence for the sex-specific surgical treatment strategy.

**Methods:**

In a prospective cohort design, participants scheduled for SG at an affiliated hospital between November 2020 and January 2022 were assessed for eligibility and allocated to the Male or Female group with a 1-year follow-up after surgery. The primary outcome was the sex difference in the weight-loss effect after SG indicated by both percentage of total weight loss (TWL%) and excess weight loss (EWL%). The secondary outcome was the analysis of sex-specific preoperative predictors of weight loss after SG based on univariate and multivariate analyses. Independent predictors were obtained to construct a nomogram model. The discrimination, calibration, and clinical utility of the nomogram were based on receiver operating characteristic curve, concordance index, calibration curve, and decision curve analysis, respectively.

**Results:**

Ninety-five male and 226 female patients were initially included. After propensity score matching by baseline body mass index (BMI), 85 male and 143 female patients achieved comparable TWL% and EWL% for 1 year after SG. For male patients, baseline BMI, area under the curve for insulin during oral glucose tolerance test, and progesterone were independent predictors of weight loss after SG. Baseline BMI, age, thyroid stimulating hormone, and Self-Rating Anxiety Scale score were independent predictors for female patients.

**Conclusion:**

No obvious sex difference is detected in the weight-loss effect after SG. Sex dimorphism exists in the predictors of weight loss after SG. Further research with long-term and a multicenter design is needed to confirm the predictive model.

## 1 Introduction

Obesity has become a global epidemic affecting more than 988 million people worldwide by 2020 (1). Bariatric metabolic surgery has been recognized as an effective and evidence-based surgical treatment for morbid obesity (2). Sleeve gastrectomy (SG) has become the most common bariatric metabolic procedure worldwide (3). However, the weight-loss effect after SG varied among patients. Insufficient weight loss and weight regain have become challenging issues (4).

Body weight can be affected by biologic, psychosocial, and behavioral factors (5). Given sex-related differences in psychosocial status, hormonal homeostasis, and body fat distribution (6, 7), responses to weight-management strategies are likely to differ by sex. For example, males were reported to get more health benefits from moderate-intensity exercise (8). However, females are significantly more successful on pharmacotherapy for weight loss (9). Moreover, there is no consensus in clinic in terms of the sex-related differences in the weight-loss effect after SG (10, 11). As mentioned above, further research is needed to illustrate the sex differences in the weight-loss effect after SG.

Preoperative prediction of weight loss is helpful not only for defining realistic expectations and maintaining motivation for patients but also for surgeons to select good candidates and reduce failures (12). However, the factors that predict weight loss following SG cannot be conclusively determined (13). Moreover, no sex-specific guidelines for surgical anti-obesity strategies have yet been proposed. Thus, it is of great importance to identify the sex-specific preoperative predictors of weight loss after SG.

Based on a prospective cohort, the present study aims to determine the sex difference in the weight-loss effect after SG indicated by both percentage of total weight loss (TWL%) and excess weight loss (EWL%), and further construct the sex-specific nomograms based on analysis of the sex-specific preoperative predictors of weight loss after SG, providing clinical evidence for the surgical treatment strategy to help achieve better weight loss.

## 2 Materials and methods

### 2.1 Study design and patients

The protocol of this study was approved by the Ethics Committee on Scientific Research of Shandong University Qilu Hospital on February 24, 2017. All data were retrieved from a prospectively collected database (SDBMSR, https://sdbmsr.yiducloud.com.cn). The conduction of this study conformed to the principles outlined in the Declaration of Helsinki. Each participant was informed in detail about the purpose, process, potential risks and benefits of the research on the day of admission. All participants signed written informed consent forms before assessment.

### 2.2 Patients and follow-up

Participants were assessed for eligibility if they were scheduled for SG between November 2020 and January 2022 at University hospital. The exclusion criteria were as follows: (1) uncontrolled mental illness such as schizophrenia, bipolar affective disorder or severe organ dysfunction such as heart failure, respiratory failure; (2) treatment with weight-loss medications; (3) SG as a revision surgery; (4) pregnancy during follow-up; and (5) incomplete follow-up data. Patients were assigned into Male and Female groups according to sex.

All patients underwent follow-up at 1, 3, 6, and 12 months after surgery. The primary outcome was weight loss after surgery evaluated by the TWL% and EWL%. The secondary outcome was the analysis of predictors of weight loss after SG in male and female patients. TWL% was calculated as weight loss/baseline weight × 100%; EWL% was calculated as [preoperative weight - postoperative weight]/[preoperative weight - 23×(body length)^2^)] × 100% (14, 15).

### 2.3 Procedure

All SG operations were performed laparoscopically by the same experienced team as reported before (16). The first step is the greater curvature was dissected free from the omentum starting 2-4 cm from the pylorus and up to the angle of His. After exposure of the left diaphragmatic crus and adequate clearance of the posterior stomach, a vertical gastrectomy was initiated from 4-6 cm proximal to the pylorus with the use of a 36-Fr bougie to create a tubular stomach. Upon discharge, the patient was given suggestions for dietary and physical activities. Dietitians recommend gradually transitioning from liquid to solid foods and achieving energy balance through higher protein, lower fat and lower carbohydrate intake. The exercise prescriber will develop an individualized exercise prescription including training frequency, intensity, time, and type.

### 2.4 Propensity score matching

Nearest-neighbor matching with caliper was used to balance baseline body mass index (BMI) between the Male and Female groups. Matching was performed with the use of a 1:2 protocol without replacement, with a caliper width equal to 0.2 (17).

### 2.5 Psychological assessment

The Chinese versions of the Self-Rating Anxiety Scale (SAS) and Self-Rating Depression Scale (SDS) were used to evaluate the psychological situation of anxiety and depression of patients the day before surgery (18, 19). A score above 50 or 53 was defined as anxiety or depression in the SAS and SDS. The levels of anxiety or depression were further classified according to the score.

### 2.6 Oral glucose tolerance test

Participants underwent a 2-hour oral glucose tolerance test (OGTT) (75 g of glucose in 250 ml water) on the second day of admission after an overnight fast. Blood samples were collected at 0, 30, 60, and 120 minutes after glucose intake. The plasma levels of glucose and insulin were determined at each time point.

### 2.7 Body composition by dual-energy X-ray absorptiometry

Visceral fat area, and body fat percentage were determined with dual-energy X-ray absorptiometry using the HOLOGIC DELPHI system with QDR software, v.11.1 (Hologic Bedford, MA, USA). The exams were whole-body scans, all of which were performed in a temperature-controlled laboratory. All operations were carried out by trained researchers according to the manufacturer’s instructional protocols. The very few cases with missing data were excluded from the analyses.

### 2.8 Biochemical analysis

Blood lipid analysis, index of thyroid function, sex hormone, liver and renal function, and the levels of plasma glucose were measured using a Roche Cobas 8000 modular analyzer system (Roche Diagnostics, IN, USA). Plasma insulin was determined by a two-site enzymatic assay using a Tosoh 2000 autoanalyzer (Tosoh Corp., Tokyo, Japan). The homeostasis model assessment of insulin resistance (HOMA-IR) index was calculated as fasting insulin (mU/mL) ×fasting plasma glucose (mmol/L)/22.5.

### 2.9 Nomogram-based prediction

Candidate clinical predictors included age, BMI, low-density lipoprotein (LDL), high-density lipoprotein (HDL), triglycerides (TG), alanine aminotransferase (ALT), thyroid stimulating hormone (TSH), area under the curve for glucose (AUC_glucose_), area under the curve for insulin (AUC_insulin_), HOMA-IR, estrogen, progesterone, androgen, prolactin, visceral fat area, body fat percentage, SAS score, and SDS score.

There is currently no clear standard for judging postoperative weight loss with respect to TWL%. In order to make more convincing predictions, patients in the Male and Female groups were ranked in descending order separately and further divided into three equal parts according to the 1-year TWL%. Univariate regression analysis was conducted between the one-third of patients with the highest TWL% and the one-third with the lowest TWL% for potential predictive factors. Those with P < 0.1 were further analyzed by multivariate regression. The results are shown as odds ratios (ORs) with 95% confidence intervals (CIs). Significant factors (P < 0.05) according to the results of the multivariate regression were considered independent predictors and used to establish the nomogram.

The area under the receiver operating characteristic (ROC) curve (AUC) and concordance index (C-index) were used to evaluate the predictive accuracy of the nomogram model. A calibration curve was used to evaluate the accuracy of the prediction. A decision curve analysis (DCA) was performed to assess the clinical usefulness of the nomogram. The nomogram was internally validated for discrimination and calibration by bootstrapping (1000 resamples).

### 2.10 Statistical analysis

Statistical analysis was performed using SPSS Statistics version 25.0 (SPSS Inc., Chicago, Illinois, US) and R version 4.2.2 (R Foundation for Statistical Computing, Vienna, Austria). Continuous variables that conformed to normal distribution were presented as the means ± SDs and compared using the independent t test; variables without normal distribution were given as median with interquartile range and compared by the Mann−Whitney U test. Categorical variables were shown as numbers with percentages and compared with the chi-squared test or Fisher’s exact test. Two-way ANOVA followed by Bonferroni’s multiple comparisons was performed to analyze weight loss over time after surgery, and the results were reported as the *^A^P* by group, *^B^P* over time, and *^C^P* due to the interaction of the two factors. The AUC of glucose and insulin during OGTT was calculated by the trapezoidal method. A *P* value <0.05 was considered to indicate significance.

## 3 Results

### 3.1 Baseline characteristics of participants

**Figure 1:**
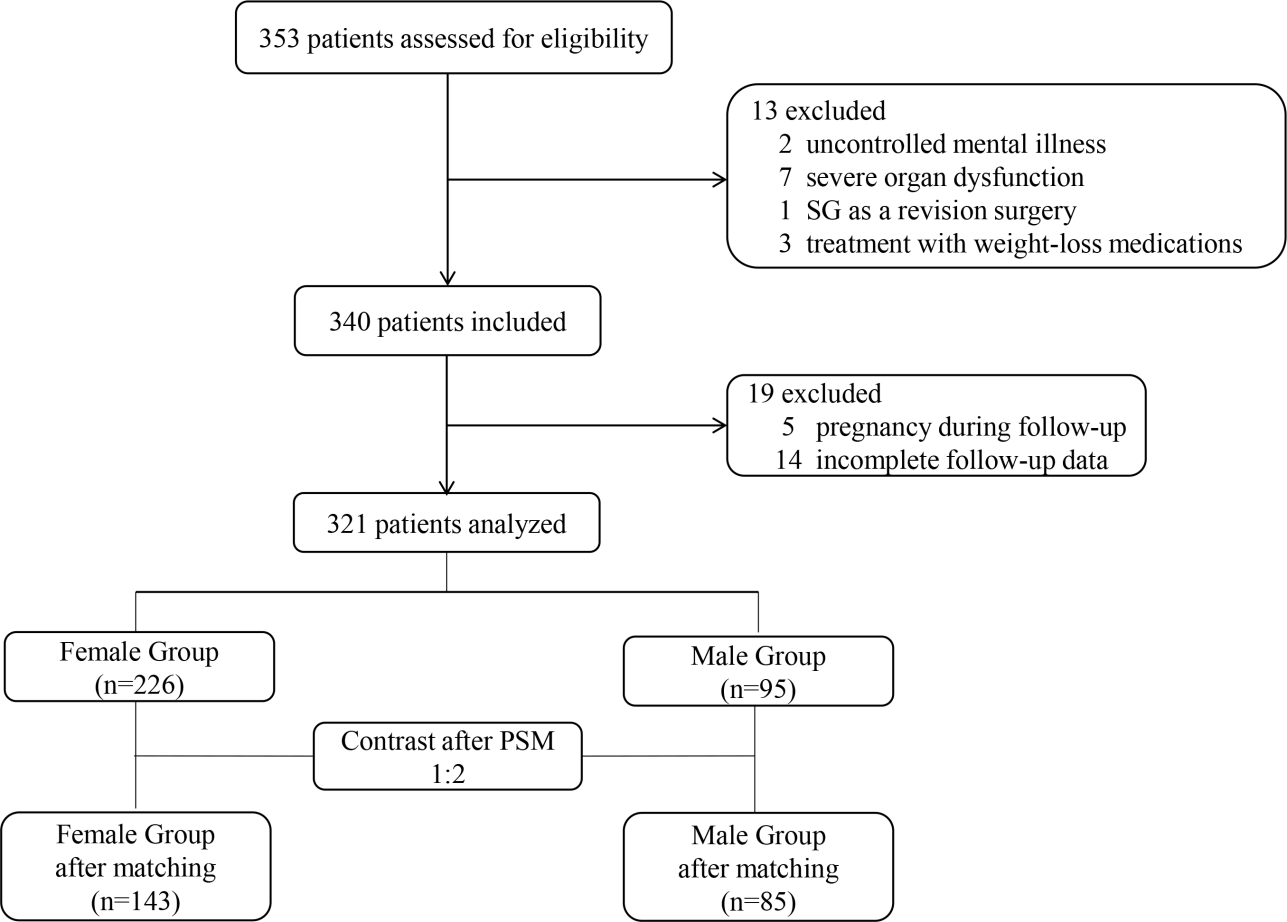
Flowchart of participants. SG, sleeve gastrectomy; PSM, propensity score matching.

From the 353 patients assessed for eligibility, 321 patients (95 male and 226 female) were included (**Figure 1**). The male patients had a significantly higher BMI (45.43 ± 8.59 vs. 40.32 ± 6.95 kg/m^2^; *P*<0.001; **Table 1**). After propensity score matching (PSM), 85 patients in the Male group and 143 patients in the Female group were preserved with balanced baseline BMI (42.48 ± 6.31 vs. 43.65 ± 6.72 kg/m^2^; *P*=0.152; **Table 2**). The obesity comorbidities were paired between the two groups. The Male group had higher levels of ALT, TG, AUC_glucose_, and androgen, while having lower levels of HDL, estrogen, progesterone, body fat percentage, SAS score and SDS score (**Table 2**).

**Table 1:** Baseline characteristics of total participants.

Index	Female (n=226)	Male (n=95)	*P* value
Age, years	32.08 ± 7.28	30.63 ± 7.74	0.112
BMI, kg/m^2^	40.32 ± 6.95	45.43 ± 8.59	<0.001
Obesity comorbidities			
T2D, n (%)	67 (29.65%)	43 (45.26%)	0.007
Hypertension, n (%)	99 (43.81%)	62 (65.26%)	<0.001
OSAHS, n (%)	131 (57.57%)	65 (68.42%)	0.079
PCOS, n (%)	81 (35.84%)	N/A	N/A
LDL, mmol/l	3.02 ± 0.70	3.01 ± 0.83	0.980
HDL, mmol/l	1.11 ± 0.21	0.99 ± 0.19	<0.001
ALT, u/l	26 (18, 53)	42 (22, 60)	0.001
TG, mmol/l	1.53 (1.08, 1.98)	1.86 (1.18, 3.20)	0.001
TSH, uIU/ml	2.00 (1.41, 2.97)	1.91 (1.40, 2.72)	0.392
AUC_glucose_	15.83 (13.48, 19.33)	16.48 (14.04, 28.56)	0.137
AUC_insulin_	144.51 (99.90, 219.74)	133.97 (67.06, 238.83)	0.202
HOMA-IR	2.50 (1.80, 3.53)	2.80 (2.00, 3.80)	0.273
Estrogens, pmol/l	176.31 (123.70, 257.61)	137.60 (107.30, 167.50)	<0.001
Progesterone, nmol/l	0.56 (0.31, 1.45)	0.48 (0.32, 0.66)	0.011
Androgen, nmol/l	1.12 (0.73, 1.68)	7.14 (5.17, 10.02)	<0.001
Prolactin, uIU/ml	257.60 (157.92, 342.48)	244.70 (170.40, 314.60)	0.453
Visceral fat area, cm^2^	189 (150, 221)	193 (163, 231)	0.080
Body fat percentage, n (%)	43.32 ± 3.91	38.65 ± 5.40	<0.001
SAS	46.68 ± 8.14	45.83 ± 7.98	0.004
Normal, n (%)	130 (57.52%)	68 (71.59%)	
Mild, n (%)	75 (33.19%)	23 (24.21%)	
Moderate, n (%)	21 (9.29%)	4 (4.2%)	
SDS	51.53 ± 9.94	48.20 ± 9.97	0.005
Normal, n (%)	130 (57.52%)	67 (70.53%)	
Mild, n (%)	61 (26.99%)	18 (18.95%)	
Moderate, n (%)	29 (12.83%)	9 (9.47%)	
Severe, n (%)	6 (2.66%)	1 (1.05%)	

Data are presented as n (%), mean ± SD, or median (25th percentile, 75th percentile).

BMI, body mass index; T2D, Type 2 Diabetes; OSAHS, obstructive sleep apnea-hypopnea syndrome; PCOS, polycystic ovary syndrome; N/A, Not Applicable; LDL, low-density lipoprotein; HDL, high-density lipoprotein; ALT, alanine aminotransferase; TG, triglycerides; TSH, thyroid stimulating hormone; AUC_glucose_, area under the curve for glucose; AUC_insulin_, area under the curve for insulin; HOMA-IR, homeostasis model assessment of insulin resistance; SAS, Self-Rating Anxiety Scale; SDS, Self-Rating Depression Scale.

**Table 2:** Basic characteristic data in the Female and Male group after PSM.

Index	Female (n=143)	Male (n=85)	*P* value
Age, years	31.45 ± 7.26	30.86 ± 7.97	0.564
BMI, kg/m^2^	42.48 ± 6.31	43.65 ± 6.72	0.152
Obesity comorbidities			
T2D, n (%)	45 (31.47%)	35 (41.18%)	0.137
Hypertension, n (%)	75 (52.45%)	54 (63.53%)	0.103
OSAHS, n (%)	86 (60.14%)	57 (67.06%)	0.296
PCOS, n (%)	51 (35.66%)	N/A	N/A
LDL, mmol/l	3.00 ± 0.70	3.04 ± 0.83	0.707
HDL, mmol/l	1.10 ± 0.19	0.99 ± 0.18	<0.001
ALT, u/l	29 (18, 56)	44 (23, 59)	0.009
TG, mmol/l	1.51 (1.08, 1.94)	2.00 (1.18, 3.36)	<0.001
TSH, uIU/ml	2.14 (1.48, 3.17)	1.86 (1.36, 2.67)	0.094
AUC_glucose_	15.88 (13.27, 19.57)	17.42 (14.23, 23.57)	0.049
AUC_insulin_	147.38 (103.73, 241.96)	126.26 (61.77, 240.10)	0.055
HOMA-IR	2.80 (1.90, 3.60)	2.80 (1.95, 3.75)	0.906
Estrogens, pmol/l	168.70 (121.33, 229.60)	130.60 (130.45, 163.00)	<0.001
Progesterone, nmol/l	0.56 (0.32, 0.97)	0.50 (0.35, 0.66)	0.044
Androgen, nmol/l	1.12 (0.73, 1.69)	7.62 (5.54, 10.49)	<0.001
Prolactin, uIU/ml	260.20 (158.20, 350.20)	240.00 (151.38, 330.15)	0.186
Visceral fat area, cm^2^	199.00 (159.00, 228.00)	193.00 (162.00, 228.50)	0.972
Body fat percentage, n (%)	44.22 ± 3.76	37.83 ± 4.89	<0.001
SAS	48.87 ± 7.89	46.09 ± 8.11	0.012
Normal, n (%)	81 (56.64%)	59 (69.41%)	
Mild, n (%)	46 (32.17%)	19 (22.35%)	
Moderate, n (%)	16 (11.19%)	7 (8.24%)	
SDS	51.57 ± 9.96	48.62 ± 9.98	0.032
Normal, n (%)	77 (53.85%)	56 (65.88%)	
Mild, n (%)	43 (30.07%)	19 (22.35%)	
Moderate, n (%)	20 (13.99%)	9 (10.59%)	
Severe, n (%)	3 (2.09%)	1 (1.18%)	

Data are presented as n (%), mean ± SD, or median (25th percentile, 75th percentile).

PSM, propensity score matching; BMI, body mass index; T2D, Type 2 Diabetes; OSAHS, obstructive sleep apnea-hypopnea syndrome; PCOS, polycystic ovary syndrome; N/A, Not Applicable; LDL, low-density lipoprotein; HDL, high-density lipoprotein; ALT, alanine aminotransferase; TG, triglycerides; TSH, thyroid stimulating hormone; AUC_glucose_, area under the curve for glucose; AUC_insulin_, area under the curve for insulin; HOMA-IR, homeostasis model assessment of insulin resistance; SAS, Self-Rating Anxiety Scale; SDS, Self-Rating Depression Scale.

### 3.2 Weight loss after SG

**Figure 2:**
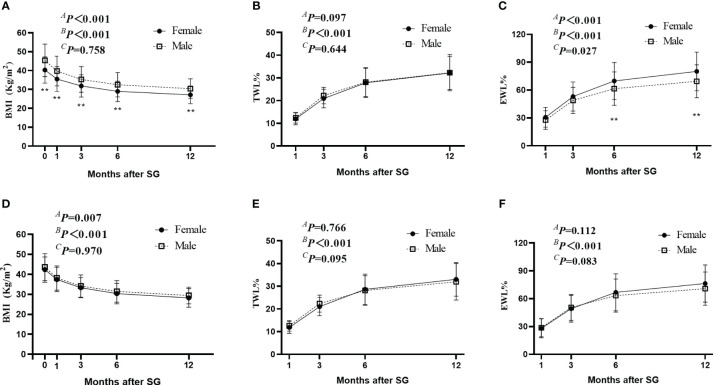
Weight-loss effect after SG. **(A–C)** indicated the change of BMI **(A)**, TWL% **(B)**, and EWL% **(C)** after SG in the original cohort before PSM. D-F indicated the change of BMI **(D)**, TWL% **(E)**, and EWL% **(F)** after SG in the Male group and Female group after PSM. *^A^P* by group, *^B^P* over time, and *^C^P* due to the interaction of the two factors. ** *P <*0.05 SG, sleeve gastrectomy; PSM, propensity score matching; BMI, body mass index; TWL%, percentage of total weight loss; EWL%, percentage of excess weight loss.

Along with the decrease in BMI, the TWL% and EWL% continued to increase for up to 1 year after SG in both the Male and Female groups (**Figures 2A–C**). For the original 321 patients before PSM, although no significant between-group difference was found in TWL% after SG (*^A^P* =0.097), the Female group showed a higher EWL% at 6 and 12 months after SG than the Male group (**Figure 2C**). However, this difference disappeared after PSM by baseline BMI. The Male and Female groups showed comparable BMI, TWL% and EWL% after SG (**Figures 2D–F**). These results suggest that for selective patients with comparable BMI, sex was not an influencing factor of weight loss for up to 1 year after SG.

### 3.3 Predictor selection and development of the nomogram

**Figure 3:**
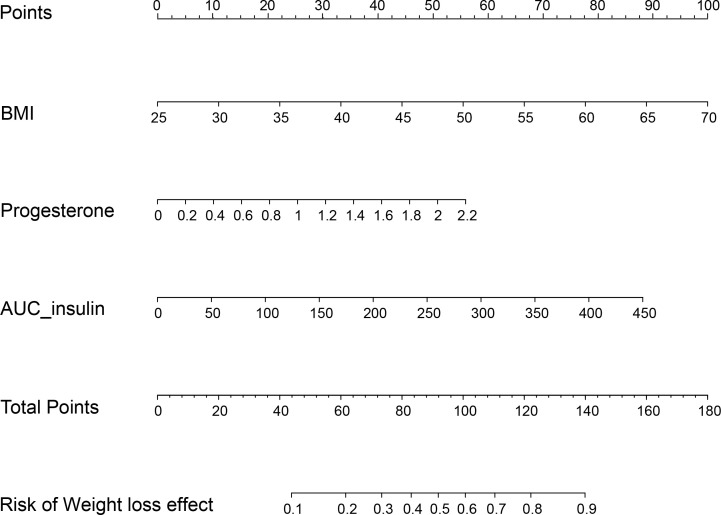
Nomogram for prediction of weight loss effect in the Male group. BMI, body mass index; AUC_insulin_, area under the curve for insulin.

**Figure 4:**
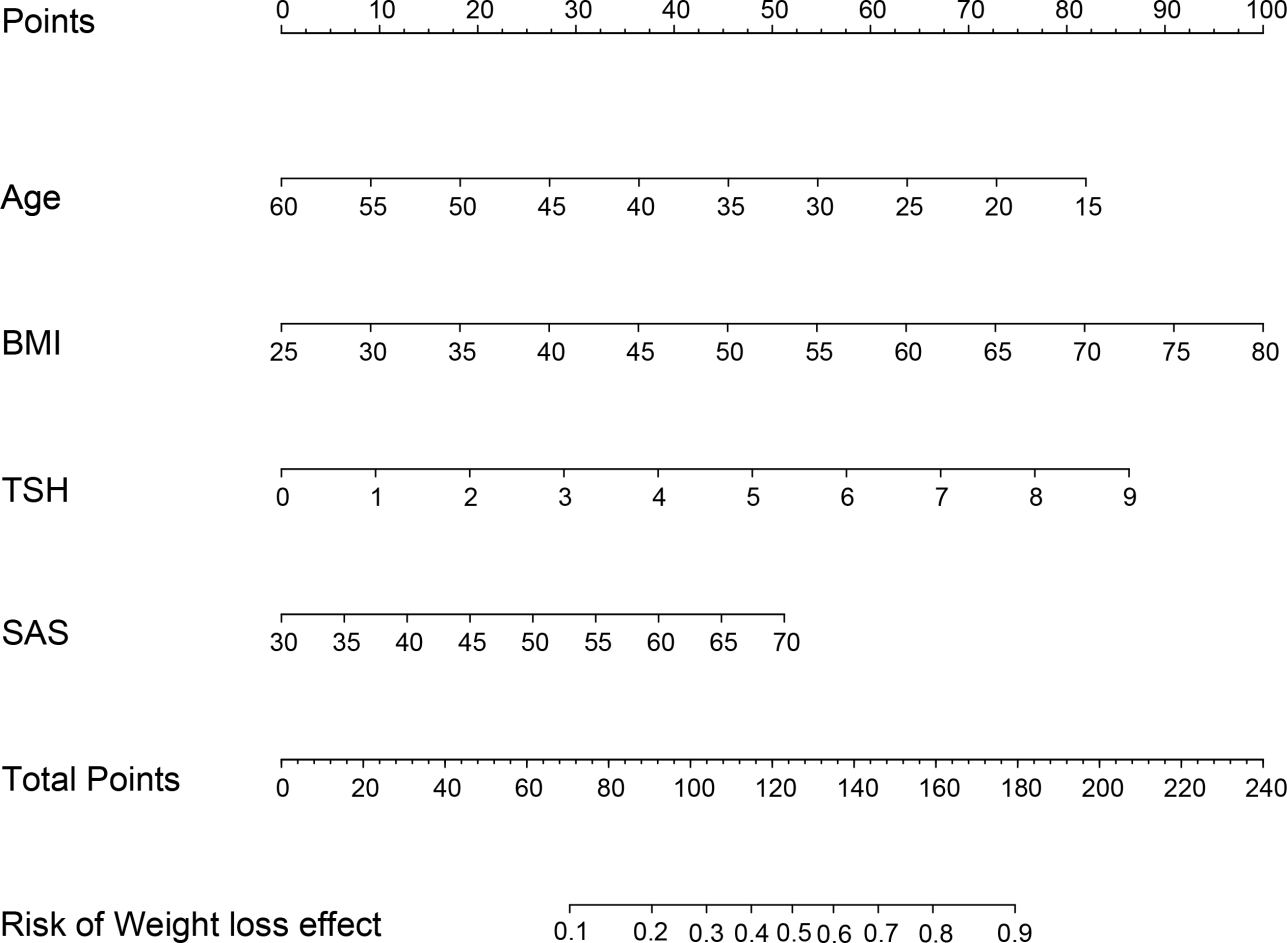
Nomogram for prediction of weight loss effect in Female group. BMI, body mass index; TSH, thyroid stimulating hormone; SAS, Self-Rating Anxiety Scale.

**Figure 5:**
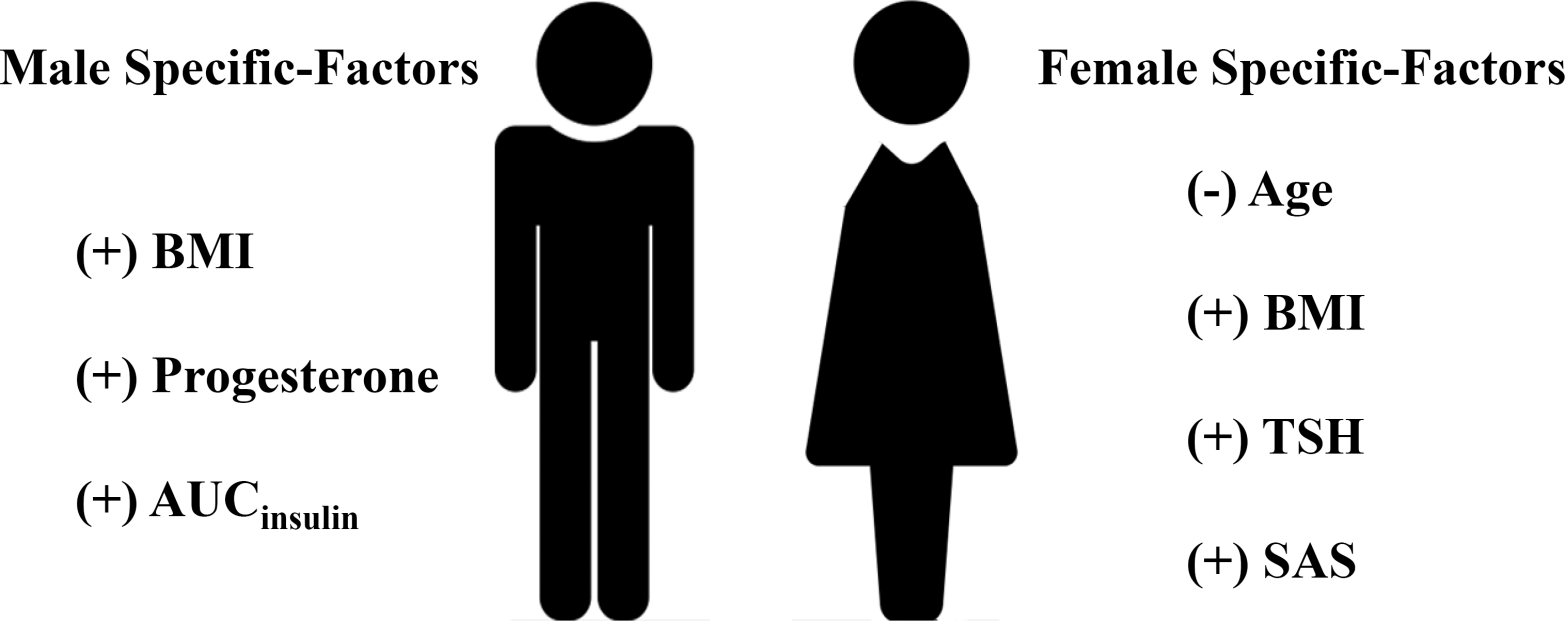
Visualization of sex-specific nomogram model of weight loss effect. (+) indicates positive correlation with weight loss effect; (-) indicates negative correlation with weight loss effect. BMI, body mass index; AUC_insulin_, area under the curve for insulin; TSH, thyroid stimulating hormone; SAS, Self-Rating Anxiety Scale.

Patients with TWL% <27.65% and >35.29% in the Male group as well as those with TWL% <29.26% and >35.49% in the Female group were included in the analysis of predictors. In the multivariate analysis, factors significantly and independently associated with the weight-loss effect in the Male group were baseline BMI [odds ratio (OR) 1.106, 95% confidence interval (CI) 1.019-1.201, *P*=0.016], AUC_insulin_ [OR 1.009, 95% CI 1.002-1.017, *P*=0.010], and progesterone [OR 3.088, 95% CI 1.031-9.256, *P*=0.044]. In the Female group, age [OR 0.929, 95% CI 0.882-0.979, *P*=0.006], baseline BMI [OR 1.076, 95% CI 1.018-1.147, *P*=0.009], TSH [OR 1.473, 95% CI 1.009-1.975, *P*=0.010], and SAS score [OR 1.053, 95% CI 1.003-1.106, *P*=0.038] were associated with the weight-loss effect after SG (**Tables 3**, **4**). These predictor factors were incorporated into the nomogram (**Figures 3**, **4**). Also, the visualization of sex-specific nomogram model of weight loss effect was illustrated in **Figure 5**.

**Table 3:** Factors associated with TWL% at 1 year after sleeve gastrectomy (male).

Factor	Univariate analysis				Multivariate analysis			
	OR	95%CI		*P* value	OR	95%CI		*P* value
Age, years	0.955	0.897	1.017	0.149				
BMI, kg/m^2^	1.131	1.046	1.222	0.002	1.106	1.019	1.201	0.016
LDL, mmol/l	1.119	0.580	2.159	0.738				
HDL, mmol/l	0.804	0.051	12.677	0.877				
ALT, u/l	1.010	0.995	1.026	0.196				
TG, mmol/l	0.617	0.407	0.935	0.023				
TSH, uIU/ml	0.848	0.539	1.335	0.477				
AUC_glucose_	0.852	0.774	0.939	0.001				
AUC_insulin_	1.012	1.005	1.019	0.001	1.009	1.002	1.017	0.010
HOMA-IR	1.512	1.039	2.199	0.031				
Estrogens, pmol/l	0.999	0.988	1.010	0.820				
Progesterone, nmol/l	2.465	1.024	5.935	0.044	3.088	1.031	9.256	0.044
Androgen, nmol/l	0.913	0.802	1.038	0.165				
Prolactin, uIU/ml	1.002	0.998	1.007	0.323				
Visceral fat area, cm^2^	1.001	0.992	1.010	0.877				
Body fat percentage, n (%)	1.215	1.075	1.374	0.002				
SAS	0.990	0.931	1.053	0.752				
SDS	0.972	0.926	1.021	0.252				

BMI, body mass index; LDL, low-density lipoprotein; HDL, high-density lipoprotein; ALT, alanine aminotransferase; TG, triglycerides; TSH, thyroid stimulating hormone; AUC_glucose_, area under the curve for glucose; AUC_insulin_, area under the curve for insulin; HOMA-IR, homeostasis model assessment of insulin resistance; SAS, Self-Rating Anxiety Scale; SDS, Self-Rating Depression Scale.

**Table 4:** Factors associated with TWL% at 1 year after sleeve gastrectomy (female).

Factor	Univariate analysis				Multivariate analysis			
	OR	95%CI		*P* value	OR	95%CI		*P* value
Age, years	0.925	0.881	0.972	0.002	0.929	0.882	0.979	0.006
BMI, kg/m^2^	1.081	1.026	1.139	0.003	1.076	1.018	1.137	0.009
LDL, mmol/l	1.093	0.702	1.702	0.695				
HDL, mmol/l	0.225	0.040	1.246	0.088				
ALT, u/l	0.995	0.986	1.004	0.265				
TG, mmol/l	0.942	0.605	1.465	0.790				
TSH, uIU/ml	1.450	1.089	1.931	0.011	1.473	1.099	1.975	0.010
AUC_glucose_	0.956	0.905	1.010	0.107				
AUC_insulin_	1.002	0.999	1.006	0.133				
HOMA-IR	1.095	0.869	1.380	0.441				
Estrogens, pmol/l	0.999	0.997	1.002	0.554				
Progesterone, nmol/l	0.997	0.920	1.080	0.939				
Androgen, nmol/l	1.506	0.956	2.370	0.077				
Prolactin, uIU/ml	1.001	0.998	1.003	0.597				
Visceral fat area, cm^2^	1.003	0.997	1.010	0.284				
Body fat percentage, n (%)	1.117	1.023	1.221	0.014				
SAS	1.049	1.003	1.097	0.036	1.053	1.003	1.106	0.038
SDS	1.023	0.990	1.058	0.175				

BMI, body mass index; LDL, low-density lipoprotein; HDL, high-density lipoprotein; ALT, alanine aminotransferase; TG, triglycerides; TSH, thyroid stimulating hormone; AUC_glucose_, area under the curve for glucose; AUC_insulin_, area under the curve for insulin; HOMA-IR, homeostasis model assessment of insulin resistance; SAS, Self-Rating Anxiety Scale; SDS, Self-Rating Depression Scale.

### 3.4 Validation and clinical utility of the nomogram for predicting

**Figure 6:**
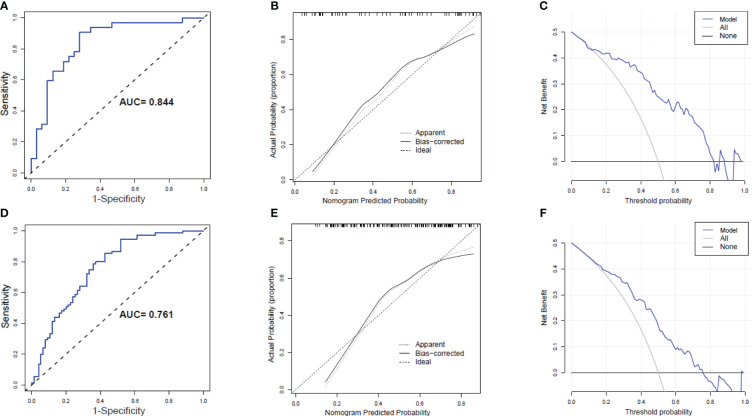
Receiver operating characteristic (ROC) curves, Calibration curves and Decision curve analysis (DCA) of the nomogram prediction in the Male group and Female group. **(A)** ROC curves in the Male group; **(B)** Calibration curves of in the Male group. **(C)** DCA in the Male group. **(D)** ROC curves in the Female group; **(E)** Calibration curves of in the Female group. **(F)** DCA in the Female group.

The AUC of the predicted nomogram was 0.844 (95% CI 0.745-0.943) in the Male group and 0.761 (95% CI 0.685-0.837) in the Female group (**Figures 6A, D**). The corrected C-index after bootstrapping was 0.818 and 0.738 in the Male and Female groups, respectively. The calibration curves of the nomogram for the predicted weight-loss effect in both groups showed good agreement between prediction and observation (**Figures 6B, E**). The DCA curve shows the obvious net benefits of the nomogram in both the Male and Female groups (**Figures 6C, F**).

## 4 Discussion

The principal findings of the current study were that although there was no sex difference in the weight-loss effect after SG, sex dimorphism exists in predictors of weight loss after SG. For male patients, baseline BMI, AUC_insulin_, and progesterone were independent predictors of weight loss after surgery. For female patients, baseline BMI, age, TSH, and SAS were independent predictors of weight loss after SG.

A marked sex disproportion exists in patients undergoing bariatric metabolic procedures. According to the Fourth IFSO Global Registry Report 2018, female patients was 73.7% among those who underwent the primary procedure (20). Controversy existed for a long time regarding whether male patients could achieve better weight-loss outcomes after bariatric surgery. A recent systematic review collected evidence on this issue and suggested that sex did not have a clear effect on the weight loss efficiency of SG (6). However, that conclusion was weakened by the high heterogeneity and low comparability of the study design, subject characteristics, and follow-up time in the 5 included studies. More specifically, 2 studies favored females, 1 favored males, and 2 showed similar weight loss between sex groups. Moreover, most of the 5 included studies did not have a baseline BMI that was balanced between the Male and Female groups. In the present study, male patients accounted for 29.56% of the entire cohort with higher BMI before surgery. Our results confirmed that for patients with comparable BMI, no sex difference was detected in the weight-loss effect after SG.

While SG is a well-established and widely performed procedure, there are still concerns that the weight-loss effect of SG cannot catch up with that of Roux-en-Y gastric bypass. The weight-loss effect of SG varied among patients and was affected by a series of factors. Identifying such factors can help surgeons make better care plans for the specific patient and decrease the numbers of revisional patients and nonresponders. Although studies are currently exploring predictors of weight loss after SG, there is no consensus among investigators (21). More importantly, there is a particular lack of sex-specific prediction models. The present study aimed to address this issue based on a prospective cohort.

The present study showed that higher baseline BMI predicted better postoperative weight loss in both sex groups. However, some studies have suggested that patients with a lower BMI tended to have more weight loss after SG (22, 23). This inconsistency could be explained by the discrepancy in the weight-loss metric used. Specifically, those other studies used EWL% or percentage of excess BMI loss (EBMIL%), whereas TWL% was used in the present study. Researchers have referred to the issue as the “Double Booby-Trap” of EWL%/EBMIL% to indicate that the conclusion would be overturned when EWL%/EBMIL% was used instead of TWL% (24). The “Double Booby-Trap” effect worsens with lower baseline BMI due to the algebraic construction of the EWL%/EBMIL%. In fact, many official academic organizations have recommended using TWL% as an alternative to EWL% (or EBMIL%) as the primary measure of weight loss (25). Studies are needed to provide more evidence on the correlation between baseline BMI and TWL% after SG. Furthermore, the necessity of PSM for baseline BMI was further demonstrated given that baseline BMI was an important predictor of weight loss in both sex groups.

In female patients, age is negatively associated with weight-loss outcomes, as has been demonstrated in a large number of studies (26–29). The basal energy expenditure has a tendency to decrease tremendously with age, switching from 60 to 70% of total metabolism around the age of 20–30 to 40% at the age of 50 (30). In addition, younger female obese patients usually have higher motivation for weight loss, probably because of their expectation of regaining self-confidence (31). Our study suggests the necessity for female patients to undergo surgery without delay because older patients need to make more efforts to achieve better weight-loss results.

Female patients with higher preoperative TSH levels achieved better weight-loss outcomes. Clinical studies have demonstrated a positive correlation between obesity and plasma TSH levels (32, 33). A previous study initially reported that the TSH level decreased significantly after SG in euthyroid patients. However, the TSH decrease was not associated with EWL% (34). The exact mechanism leading to a decrease in TSH following SG is not clear. The main explanation, suggested by several studies, is related to a decrease in leptin levels following surgery, which was produced by adipocytes and was shown to have a stimulatory effect on thyroid activity. Muraca et al. reported that baseline TSH levels had no association with weight loss after SG (35). However, as most studies assessing the efficiency of SG failed to report results by sex, there is still a lack of direct evidence in terms of the correlation between baseline TSH level and the weight-loss effect after SG in female patients. The predictive value of baseline TSH needs to be confirmed by further studies.

The rate of psychological behavior abnormalities among obese people who are willing to undergo bariatric surgery is as high as 70% (36). In the present study, preoperative anxiety was another positive predictor of weight loss in female patients. These results are believed to be related to the increased adherence to the postoperative instructions and were consistent with those of previous studies (37, 38). A study published in 2022 revealed an interesting trajectory in which patients with higher levels of anxiety lost the most weight 12 months after bariatric surgery but tended to regain more weight 30 months after surgery (39). The mechanisms by which anxiety contributes to this trajectory should be examined in future research. Our study suggested that female patients are more susceptible to the impact of anxiety. For female patients, the identification and intervention of psychological disorders, especially those associated with anxiety, is of vital importance.

In the present study, higher levels of progesterone predicted better weight-loss outcomes in male patients. Although little is known about the physiology, endocrinology, and pharmacology of progesterone in male, it has been found that progesterone can bind to certain receptors in adipose tissue and regulate lipoprotein lipase, thereby increasing fat accumulation (40). In addition, progesterone can synergize with estrogen to reduce lipolysis and promote fat accumulation (41). Unfortunately, there is limited evidence on the alteration of progesterone after SG in male patients, let alone its effect on or correlation with weight loss. A meta-analysis published in 2019 reported the impact of bariatric surgery on male sex hormones (42). However, no progesterone data were included. Based on our prediction model, progesterone may play specific physiological and pathophysiological roles in the weight-loss effect after SG in male. Further studies are needed.

Insulin resistance is the fundamental pathophysiological change in obesity. An increase in body weight induces insulin resistance and compensates for hyperinsulinemia, resulting in fat accumulation and metabolic disorders (43). AUC_insulin_ represents the total amount of insulin secretion after oral glucose load and can indicate insulin resistance to some extent (44). Our previous study confirmed the great effect of SG on the remission of insulin resistance (16), and the decrease in AUC_insulin_ after SG was related to weight loss after surgery. Therefore, patients with higher levels of AUC_insulin_ are likely to benefit more from surgery to achieve better weight loss. The present study confirmed that AUC_insulin_ was another positive predictor of weight loss after SG in male patients.

The major strength of the present study is that the sex-specific preoperative predictors of weight loss after SG were explored from a comprehensive set of clinical variables based on a prospective cohort. Our study has several limitations. First, the 1-year follow-up time was not long enough. However, the period of weight loss after SG is approximately 1 year, and the longer-term outcome may be affected by more factors in addition to surgery. Second, due to the single-center design and limited sample size, the predictive model was validated internally by bootstrapping and needs to be further confirmed by external validation.

## 5 Conclusion

The sex was not an influencing factor of weight loss for up to 1 year after SG. However, sex dimorphism still exists in terms of the preoperative predictors of weight loss after SG. For different sex groups, there could be differences in the focus of preoperative evaluation in regard to the weight-loss effect. Research with multi-centered design and long-term follow-up as well as the mechanism of TSH affecting the weight loss effect are of great significance in the future.

## Data availability statement

The raw data supporting the conclusions of this article will be made available by the authors, without undue reservation.

## Ethics statement

The studies involving humans were approved by Ethics Committee on Scientific Research of Shandong University Qilu Hospital. The patients/participants provided their written informed consent to participate in this study.

## Author contributions

JS: Data curation, Formal Analysis, Writing – original draft. TZ: Data curation, Formal Analysis, Writing – original draft. SX: Data curation, Formal Analysis, Writing – original draft. TL: Supervision, Validation, Writing – original draft. YZ: Supervision, Validation, Writing – original draft. XH: Funding acquisition, Writing – review & editing, Writing – original draft. SL: Funding acquisition, Writing – review & editing, Writing – original draft.
